# Feasibility of using postal and web-based surveys to estimate the prevalence of tuberculosis among health care workers in South Africa

**DOI:** 10.1371/journal.pone.0197022

**Published:** 2018-05-10

**Authors:** Pinky N. Manana, Lazarus Kuonza, Alfred Musekiwa, Hendrik Koornhof, Ananta Nanoo, Nazir Ismail

**Affiliations:** 1 South African Field Epidemiology Training Programme, National Institute for Communicable Diseases, Johannesburg, South Africa; 2 School of Health Systems and Public Health, Faculty of Health Sciences, University of Pretoria, Pretoria, South Africa; 3 Centre for Tuberculosis, National Institute for Communicable Diseases, Johannesburg, South Africa; Western University, CANADA

## Abstract

**Introduction:**

Health Care Workers (HCWs) are among the highest risk groups for contracting tuberculosis (TB), which is ranked the third most common occupational health disease in South Africa. Little is known about the true extent of the burden of TB among South African HCWs and current surveillance approaches are inadequate. The study aimed to determine the feasibility of using postal and web-based surveys accessed through registries of registered professionals to estimate the prevalence of TB among HCWs in South Africa.

**Materials and methods:**

We conducted a cross sectional survey on a sample of professional nurses and doctors (general practitioners) registered on the Medpages database platform; a subscription based registry for practising health care professionals. The survey included professionals who were actively involved in the clinical management of patients, either in public or private health care facilities. The paper based survey, including pre-paid return envelopes, was distributed via the post office and web-based surveys were distributed via e-mail through a hyperlink. Descriptive statistics were used to summarize the data and the Chi-square test to determine associations between categorical variables. Active TB was defined as any history of TB

**Results:**

Out of a total of 3,400 health care professionals contacted, 596 (18%) responses were received: 401 (67%) web-based and 195 (33%) postal. A significantly higher percentage of complete forms were from postal compared to web-based (97% [189/195] versus 87% [348/401], p<0.001). Younger (<60 years) professionals were more likely to use the web-based compared to postal (87% [236/270] versus 71% [134/189], p<0.001). Overall, the prevalence of active TB infection was 8.7%, (95%CI: 6.3%–11.7%) and there was no difference observed between doctors and nurses (10.8% [18/167] versus 7.5% [22/292], p = 0.236).

**Conclusion:**

This novel approach demonstrated the feasibility of using an existing registry of professionals to conduct surveys to estimate the prevalence of TB. Our findings showed a high TB prevalence; however the estimate might have been biased by the low response rate. Further research to optimise our approach could lead to a viable option in improving surveillance among health care professionals.

## Introduction

Tuberculosis (TB) is caused by a bacterium known as *Mycobacterium tuberculosis* and is a life threatening and contagious disease [1]. TB can be diagnosed early through active screening of people at high risk of the disease, thus affording patients the benefit of early treatment with early containment of TB transmission. Effective treatment with anti—TB drugs according to the approved anti—TB guidelines is available [2]. Through effective TB diagnosis and treatment, an estimated 37 million lives were saved worldwide during 2000–2013 [3].

Worldwide, over 59 million workers are employed in health care facilities and have the potential to be exposed to daily health and safety hazards, including TB, Human Immunodeficiency Virus (HIV) and hepatitis [4]. HCWs are one of the groups at high risk for developing active TB. Other groups at increased risk include those living with HIV [5], miners [6], prison inmates [7], people living in informal settlements [8] and children [9]. TB transmission is a known risk in health care settings to both the HCWs and patients [10], regardless of the local incidence of TB as reported worldwide [11]. In South Africa (SA), TB is the third most commonly reported disease among occupational health diseases [12, 13], following noise—induced hearing loss and dermatitis.

According to HCW surveys that have been done in high income countries using either postal, web–based or telephonic studies; response rates have varied. A study done in the United States of America (USA) by La Vella et al [14] using the postal approach attained a response rate of 25%. A telephone survey that was conducted from March 2011 to March 2013 by Lowe S et al [15] in the USA, gave a response rate of 55%. A study conducted in the USA through the Medscape and Survey Sport panel [16] using a web-based approach gave a response rate of 96%. A similar study conducted in Canada had a response rate of 79% [17].

Little is known about the extent of the burden of TB among HCWs in SA and this is highlighted by a recent systematic review article by Grobler et al. 2016 [18]. Although in SA, a unique opportunity for TB screening exists in the workplace during pre—employment and annual medical examinations, as well as exit medical examinations, there is currently no operational national surveillance system to monitor TB among HCWs [19].

The aim of this study was to determine the feasibility of estimating the prevalence of TB among HCWs using postal and web-based surveys accessed through existing registries of health professionals in SA.

## Materials and methods

Three types of health professional registries were explored namely; the mandatory registries (HPCSA—Health Professions Council of South Africa and SANC—South African Nursing Council); the voluntary registries (SAMA—South African Medical Association and DENOSA—Democratic Nursing Organisation of South Africa) and Commercial registries (e.g. Medpages). Discussions with the mandatory and voluntary registries to provide access to the database for the study was unsuccessful with the common concern related to the protection of personal information (POPI Act 2013) [20]. Thus, the available commercial registry held by Medpages was approached and permission received.

A cross-sectional survey was carried out among HCWs between July and September 2016, using postal and web-based platforms. Medpages is a subscription based commercial directory of contact information for health care professionals and health care organizations/institutions in Southern Africa, covering both public sector and private sector health care provider [21]. The Medpages database contains records of more than 250 000 health care providers in SA (including about 12 255 general medical practitioners and 12 205 nurses), from both the public and private sectors [21].

In this study, the Medpages database was used as a platform to access the HCWs that were included in the survey. All general practitioners and professional nurses registered on the databases that had information on their cell phone number, e-mail and physical address were included in the sampling frame. As the database generally over represents the private sector (more than the public sector), all the HCWs from the public sector that met the inclusion criteria were included in the survey and consecutive private sector HCWs were added beginning with the most recently updated entry and registration, until the required sample size was reached.

A self—administered questionnaire used for data collection was divided into four sections: demographic data, medical history, treatment and general information. Assessing the prevalence of tuberculosis the following set of questions were used with “yes”, “no” options including: Have you ever been diagnosed with TB?; Have you been treated for TB?; What time of TB were you diagnosed with options of “pulmonary tuberculosis” or “extra-pulmonary tuberculosis”. A paper form of the questionnaire (including a pre-paid return envelope) was sent using the South African Post Office standard mail service and a hyperlink to the web-based survey was sent via e-mail using Qualtrics survey tool (Provo Utah USA). Each survey (both the postal and web-based), was allocated a different unique number linked to the database to allow us to identify individual responses and tracking of postal and web-based survey. Reminders were sent via short message service (sms) and e-mail two weeks after the start of the survey and two weeks before the close of the survey period.

## Data management and statistical analysis

All completed questionnaires were reviewed for completeness and correctness. Forms missing information on key variables (occupation and sector) were excluded. The data was exported from the Qualtrics survey tool (Provo Utah USA) into Microsoft Office Excel 2007. Descriptive statistics were used to summarize the data and further statistical analysis was done using STATA version 13 (StataCorp LP). The association between categorical variables was assessed for statistical significance using the Chi—square test. P-values less than 0.05 were considered statistically significant. The response rate was calculated by dividing the number of responses by the total number of questionnaire sent out and the prevalence of TB was calculated by dividing the number of participants who had history of TB by the total number included in the analysis.

## Ethical considerations

We obtained ethical clearance from the Faculty of Health Sciences Research Ethics Committee of the University of Pretoria (415/2015). Permission to access the Medpages database was obtained from Medpages. HCWs provided informed consent prior to the completion of the questionnaire, by ticking the “yes” button on the questionnaire.

## Results

**Fig 1:**
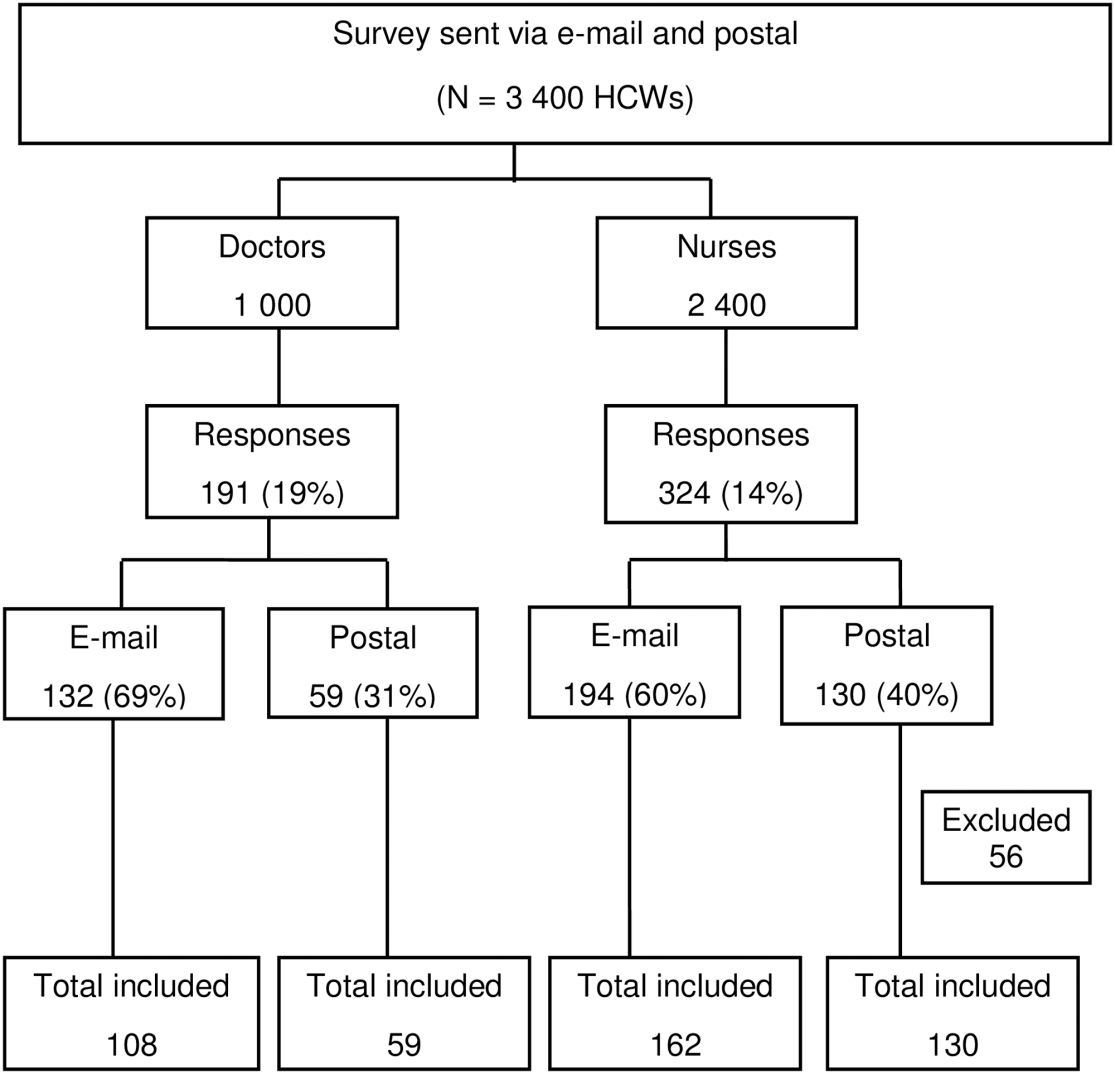
Flow diagram for study participants July—September 2016, South Africa.

A total of 3,400 HCWs were invited to complete the survey (Fig 1). Of these, 1000 (29%) were sent to doctors and 2 400 (71%) were sent to nurses, both via post and e-mail. Of the 1 000 surveys that were sent to doctors, 191 (19%) responses were received, with 132 (69%) from e-mail and 59 (31%) from postal survey. Of the 2 400 surveys that were sent to nurses, 324 (14%) responses were received, with 196 (60%) from e-mail and 130 (40%) from postal survey. An overall number of postal surveys received were 195 (33%) and overall e-mail responses were 401 (67%). A total of 81 (13.5%) forms (6 postal and 75 e-mail) were excluded because of incomplete information. The completeness of the forms varied between the two communication channels: 97% (189/195) for postal and 87% (326/401) for web-based (p<0.001). A total of 459 (14%) HCWs responses were complete and included for further analysis.

### Characteristics of study participants

Of the 459 HCWs included in this analysis, 74% (342/459) were females, with a median age of 51 years (IQR: 42–58). Medical doctors accounted for 36% (167/459) and 64% (292/459) were nurses. A total of 56% (260/459) of the HCWs were working in the public sector and the most commonly reported workplaces were the primary health care clinics 27% (122/459) and academic/ provincial hospitals 19% (86/459) (Table 1).

**Table 1:** Characteristics of the study participants, July—September 2016, South Africa.

Characteristics	n = 459	%
Gender*		
Male	116	25
Female	342	74
Age (in years)*		
18–29	5	1
30–44	134	29
45–59	240	52
60+	79	17
**Occupation**		
Doctors	167	36
Nurses	292	64
Work sector*		
Public	260	57
Private	156	34
Both	42	9
**Workplace**		
Academic/ provincial hospital	86	19
District hospital	58	13
Primary / community health care	122	27
Private hospital	23	5
Private clinic	29	6
Own practice	51	11
Other	90	19
Province*		
Gauteng	122	26
Mpumalanga	21	5
Limpopo	36	8
KwaZulu-Natal	66	14
North West	17	4
Free State	21	5
Northern Cape	20	4
Western Cape	89	19
Eastern Cape	54	12
Unknown	13	3

*unknown responses not recorded

### Methods of communication

The highest responses were received from the web-based survey (59%; 270/459). More doctors (65%; 167/459) responded via web-based survey compared to nurses (55%; 292/459), although this was not statistically significant (p = 0.054). A significant association was observed between age and methods of responses (p = 0.001), with younger age-groups preferring web-based and the older age-groups preferring the postal survey (Table 2).

**Table 2:** Preferred method of response to the survey among study participants, July—September 2016, South Africa (N = 459).

Factors	Web-based n = 270 (%)	Postal n = 189 (%)	p-value
**Occupation**			
Doctors	108 (65)	59 (35)	0.054
Nurses	162 (55)	130 (45)	
**Gender**			
Male	70 (60)	46 (40)	0.724
Female	200 (58)	142 (42)	
**Age**			
18–29	5 (100)	0 (0)	<0.001
30–44	96 (72)	38 (28)	
45–59	135 (56)	105 (44)	
60+	33 (43)	46 (57)	
**Work sector**			
Public	151 (58)	109 (42)	0.877
Private	93 (60)	63 (40)	
Both	26 (62)	17 (38)	
**Work place**			
Academic/ provincial hospital	54 (63)	32 (37)	0.668
District hospital	31 (53)	27 (47)	
Primary/ community health care clinic	67 (56)	55 (44)	
Private hospital	16 (70)	7 (30)	
Private clinic	17 (59)	12 (41)	
Own practice	27 (53)	24 (47)	

### Prevalence of TB among health care workers

Of the 459 records included in the analysis, 40 (8.7%) reported having been diagnosed and treated for TB previously. Overall prevalence of TB in this study was 8.7% (95%CI: 6.3%–11.7%). The prevalence among doctors was 11% (18/167) and among nurses was 7% (22/292). However, this difference was not statistically significant (p = 0.236). The majority of the HCWs with TB history, 63% (32/40), were diagnosed in the public sector. We also observed that 10% (4/40) of the HCWs with TB history had been diagnosed with drug resistant TB; all four were doctors. All those previously diagnosed with TB completed a course of TB treatment for the prescribed duration period ranging from 6 to 24 months.

Approximately 70% (28/40) of the HCWs diagnosed previously with TB were from Gauteng (11), Western Cape (7) and KwaZulu-Natal (10). TB among HCWs across the three provinces was 9%, 8% and 15% respectively. Of the HCWs with a previous history of TB, 58% (23/40) had worked in health care services for more than twenty years. Furthermore, 25% (10/40) had spent approximately 0 to 10 hours per week in clinical activities. However, there was no association between occupation, age, province, length of employment, and duration in clinical activities with a history of having TB (Table 3).

**Table 3:** Prevalence of TB among the study participants, July—September 2016, South Africa.

Variables	All N = 459	History of TB n = 40	Prevalence %	p-value
**Occupation**				
Doctors	167	18	11	0.236
Nurses	292	22	7	
**Age**				
18–29	5	1	20	0.538
30–44	134	12	10	
45–59	240	18	7	
60+	79	9	11	
**Gender**				
Male	116	12	10	0.477
Female	342	28	8	
**Province**				
Gauteng	122	11	9	0.604
Mpumalanga	21	1	5	
Limpopo	36	1	3	
KwaZulu-Natal	66	10	15	
North west	17	2	12	
Free state	21	3	14	
Northern Cape	14	1	7	
Western Cape	89	7	8	
Eastern Cape	54	4	7	
**Length of employment**				
0–2 years	2	1	50	0.112
3–5 years	13	3	23	
6–10 years	53	3	6	
11–15 years	56	6	11	
16–20 years	60	4	7	
21+ years	275	23	8	
**Duration in clinical activities**				
0–10 hours	79	10	13	0.679
11–20 hours	37	3	8	
21–30 hours	63	6	9	
31–40 hours	81	6	7	
41–50 hours	92	4	4	
51–60 hours	29	3	10	
61–70 hours	13	2	15	
70+ hours	64	6	9	

## Discussion

Estimating the prevalence of TB among HCWs using both postal and web-based surveys was feasible. Although the response rate was low, extrapolating from the responses we found that postal surveys were preferred by older (≥60 years) age groups and the web-based surveys were preferred by younger (<60 years) age groups. The survey was relatively easy to employ and was completed in a period of two months.

This was the first study in South Africa to directly compare a web based survey to postal based survey among HCWs and we found that more than half of the HCWs responded via web-based survey. A health related postal survey was conducted in the USA [14] and showed only a 25% response rate. This contrasts with another health related web-based survey using the Medscape and Survey Sport panel that had a response rate of 96% [16]. However, even between web-based surveys, response rates can vary as shown by two surveys conducted in Canada, done through the organisation affiliates. A reported response rate of 79% [17] was received for the one, while only 227 completed responses were received for the other with unknown response rate [22].

Age was significantly associated with the method of response, with older HCWs preferring postal, while younger HCWs preferred the web-based survey. This is an important determinant on how future surveys should be conducted. However, one third of the HCWs aged 30 years and above used the postal survey; therefore if mixed age groups are surveyed both options should be provided.

Our study had more responses from the web-based survey. The explanation could be that the web-based surveys were perceived as more timely and easy to complete. Several other studies using web-based survey also achieved high response rates, ranging from 75% to 96% [16, 17, 23]. However these were in high income countries where availability and use of web-based systems are widely utilised, which is not the norm in SA.

The overall prevalence of TB in our study was 8.7%, which is much higher than previously found in the general SA population. A study in four provinces of SA by the University Research Company (URC) in 2008, determined the TB burden among HCWs to be 2% [24] while a more recent study by O’Hara et al. 2017 reported an incidence of 1 496 per 100 000 population or 1.5% [25]. As we determined prevalence not incidence it is expected that the former would be higher. However, the higher prevalence may also be driven by a result of selection bias in the current study, where people with TB may be more motivated to complete the survey and share their experience. However, a study done in Mozambique during April 2008 –April 2010 identified a prevalence of 21% (42/201) among HCWs in an occupational health program [23]. Another study in KwaZulu-Natal from January 2006 to December 2010, using record reviews in three district hospitals, found a prevalence among HCWs of 9% (112/1,313) [26], which is the same as the current study.

We did not find any association between age, occupation, length of employment, sector and workplace with history of TB. However, other studies found an association between older age [27, 28], being a doctor or a nurse compared to students, other professions and administration staff [27–29] and longer duration of employment [28].

This study had several challenges and limitations. Access to the database from the legislated professional councils for nurses and doctors in SA was denied with reference made to protection of personal information (POPI) Act 2013 [20].This ban included anonymised data such as the required postal and e-mail contact details. As a result, only the commercially available Medpages database was utilised for the survey, and this captures more private sector HCWs than the public sector HCWs. Another limitation was a possible selection bias towards those who had a previous history of TB. This may have artificially inflated the identified prevalence, as it is probable that HCWs with previous TB would be more likely to respond to the survey. Secondly, the anonymity of the study limited our access to information that could be used to follow up on incomplete forms and non-responders. Thirdly, as random sampling was not done due to use of commercial database, there could have been a sampling bias and thus generalization of our findings has to be made with caution. The analysis was not adjusted for potential clustering, stratification, or non-response. Additionally, no verification of information by medical records was done as information was based purely on self-reporting. Lastly, there is a possibility that HCWs could have completed both the postal and web-based survey. Unique numbers were allocated to detect such occurrences; however, respondents in the main did not capture this information in their responses.

Despite the low response rate, our findings do suggest that these HCWs were more confident in reporting the TB status due to anonymity of the survey design as evidenced by the higher prevalence. However, the low response rate diminished representativeness and the reduced statistical power limited our ability to show any association between our identifiable factors. Imputation was considered, but not used, due to the high level of missing data.

## Conclusion

In conclusion, although this was the first study in SA to use postal and web–based survey approaches to estimate the prevalence of TB among HCWs registered on a database of professionals, we were able to show that the approach was feasible. Web-based surveys are useful tools for timely and efficient responses, however, additional measures need to be employed in order to increase the response rate and encourage participation through awareness campaigns and incentives. Overall the prevalence of TB was high among HCWs in this survey. Future studies using our approach conducted under the umbrella of the professional bodies are likely to yield more robust data to estimate the true burden. The ease of the survey application could also allow for annual surveys to monitor trends of TB prevalence in South Africa.

We recommend that professional bodies should consider our approach and conduct the surveys annually to generate essential data to promote health protection for HCWs. Further qualitative research is required to understand barriers to participation and increase response rates.

## Supporting information

S1 File Data set HCW survey. (XLSX)
